# The prognostic impact of NF-*κ*B p105, vimentin, E-cadherin and Par6 expression in epithelial and stromal compartment in non-small-cell lung cancer

**DOI:** 10.1038/sj.bjc.6604713

**Published:** 2008-10-14

**Authors:** S Al-Saad, K Al-Shibli, T Donnem, M Persson, R M Bremnes, L-T Busund

**Affiliations:** 1 Institute of Medical Biology, University of Tromso-Norway, Tromso, Norway; 2 Department of Pathology, University Hospital of North Norway, Tromso, Norway; 3 Department of Pathology, Nordland Central Hospital, Bodo-Norway, Norway; 4 Institute of Clinical Medicine, University of Tromso-Norway, Tromso, Norway; 5 Department of Oncology, University Hospital of North Norway, Tromso, Norway

**Keywords:** NSCLC, vimentin, par6, epithelial, stromal, disease-specific survival

## Abstract

Vimentin, nuclear factor-*κ*B (NF-*κ*B) p105, fascin, E-cadherin, TGF-*β*, Par6 and atypical PKC are molecular markers that play an important role in cell differentiation. Herein, we investigate their prognostic impact in primary non-small-cell carcinoma (NSCLC). Tumour tissue samples from 335 resected patients with stage I–IIIA were used. Tissue microarrays were constructed from duplicate cores of both neoplastic cells and stromal cells and were immunohistochemically evaluated. In univariate analyses, high tumour epithelial cell expressions of NF-*κ*B p105 (*P*=0.02) and E-cadherin (*P*=0.03) were positive prognostic indicators for disease-specific survival (DSS), whereas high tumour epithelial cell expression of vimentin (*P*=0.001) was a negative prognostic indicator. High expression of NF-*κ*B p105 (*P*=0.001) and Par6 (*P*=0.0001) in the stromal compartment correlated with a good prognosis. In multivariate analyses, the tumour epithelial cell expression of NF-*κ*B p105 (*P*=0.0001) and vimentin (*P*=0.005) and the stromal cell expression of NF-*κ*B p105 (*P*=0.007) and Par6 (*P*=0.0001) were independent prognostic factors for DSS. High expression of NF-*κ*B p105 and low expression of vimentin in tumour epithelial cells are independent predictors of better survival in primary NSCLC. In stromal cells, high expressions of NF-*κ*B p105 and Par6 are both favourable independent prognostic indicators.

Regarded as a rare malignancy as recently as 1945, lung cancer is now the most common cause of cancer death worldwide, with an estimated annual incidence of more than 1.2 million cases and a mortality of more than 1.1 million cases per year (Jemal *et al*, 2002). Despite recent advances in chemotherapy, radiation therapy and surgery, the overall survival for lung cancer patients is still less than 15%. This is particularly related to the large population with advanced stage of disease at diagnosis and the poor treatment effects in metastatic disease (Toloza and D’Amico, 2005). Non-small-cell carcinoma (NSCLC) accounts for at least 80% of all lung tumours (Ihde and Minna, 1991) and is subdivided into four major histological groups: squamous cell carcinoma (SCC), adenocarcinoma (AC), bronchioalveolar carcinoma (BAC) and large-cell carcinoma (LCC) (Travis *et al*, 2004).

A major part of patients diagnosed with cancer die as a result of cancer metastases resistant to conventional therapy (Fidler, 2002), yet the mechanisms regulating tissue changes from benign to invasive and finally to metastatic carcinoma remain fairly vague (Chaffer *et al*, 2006). A better understanding of these mechanisms will yield major contributions for improving both diagnostic and therapeutic procedures. A range of mechanisms used by oncogenes or tumour suppressor genes during malignant transformation has been revealed. Some of these are accompanied by morphological changes.

Herein, we investigate the expression of a panel of seven markers associated with tumorigenesis in NSCLC both in epithelial tumour cells and in tumour stroma. Our study included the phosphorylated nuclear factor-*κ*B (NF-*κ*B) subunit p105 (NF-*κ*B p105), vimentin, E-cadherin, atypical PKC (aPKC), Par6, fascin and transforming growth factor-*β* (TGF-*β*). Their prognostic value has been assessed using high-throughput tissue microarray (TMA) analyses.

## Patients and methods

### Patients and clinical samples

Primary tumour tissues from anonymised patients diagnosed with NSCLC pathologic stage I–IIIA (Greene *et al*, 2002) at the University Hospital of North Norway (UNN) and Nordland Central Hospital (NLSH) from 1990 through 2004 were used in this study. In total, 371 patients were registered from the hospital database. Of these, 36 patients were excluded from the study due to (i) radiotherapy or chemotherapy before surgery (*n*=10); (ii) other malignancies within 5 years before the NSCLC diagnosis (*n*=13); and (iii) inadequate paraffin-embedded fixed tissue blocks (*n*=13). Thus, 335 patients with complete medical records and adequate paraffin-embedded tissue blocks were eligible.

This report includes follow-up data as of 30 September 2005. The median follow-up was 96 (range 10–179) months. Complete demographic and clinical data were collected retrospectively. Formalin-fixed and paraffin-embedded tumour specimens were obtained from the archives of the Departments of Pathology at UNN and NLSH. The tumours were staged according to the International Union Against Cancer's TNM classification and histologically subtyped and graded according to the World Health Organization (Travis *et al*, 2004). Regarding N status, ipsilateral peribronchial or hilar nodes and intrapulmonary nodes are defined as N1, whereas N2 includes ipsilateral mediastinal or subcarinal nodes.

### Microarray construction

All lung cancer cases were histologically reviewed by two pathologists (SAS and KAS), and the most representative areas of viable invasive carcinoma tissue (epithelial cells) and surrounding tumour stroma from central parts within the tumour were carefully selected and marked on the haematoxylin and eosin slides and sampled for the TMA blocks. The TMAs were assembled using a tissue-arraying instrument (Beecher Instruments, Silver Springs, MD, USA). The detailed methodology has been reported earlier (Bremnes *et al*, 2006).

TMA represents a practical tool for studying a large number of biological markers that may have significant therapeutic effects. Studying whole tissue sections is, however, still needed to increase the value of the TMA results before therapeutical implementation. Briefly, we used a 0.6-mm diameter stylet, and the study specimens were routinely sampled in duplication from epithelial cancer cells and from tumour-surrounding stroma intervening malignant epithelial areas. Normal lung tissue localised distant from the primary tumour were used as negative controls.

To include all core samples, eight tissue array blocks were constructed. Multiple 5-*μ*m sections were cut with a Micron microtome (HM355S) and stained by specific antibodies for immunohistochemistry (IHC) analysis.

### Immunohistochemistry

**Figure 1:**
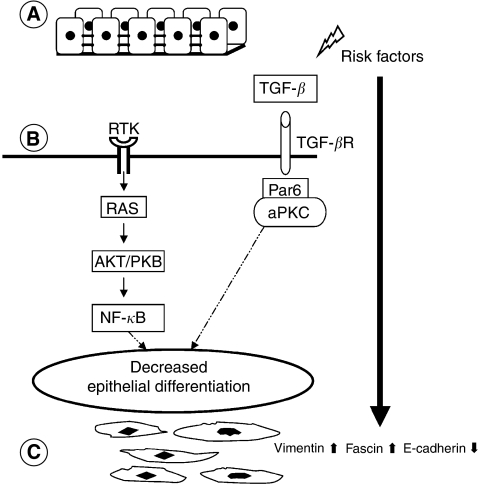
Simplified schematic illustration of signals regulating cell differentiation. (**A**) Polarised normal epithelium cells with tight junctions between cells resting on the basement membrane. (**B**) Different risk factors (including cigarette smoke, toxins and genetic factors) can cause activation of different oncogenes, which regulate cell differentiation. Activation of receptor tyrosine kinases (RTKs) is known to play a role in inducing mesenchymal phenotype. (**C**) Unpolarised spindle-shaped epithelial cells with loss of tight junctions and upregulation of mesenchymal markers like vimentin and fascin. TGF-*β*=transforming growth factor-*β*; TGF-*β*R=transforming growth factor-beta receptor; RAS=rat sarcoma oncogene; NF-*κ*B=nuclear factor-*κ*B; AKT/PKB=AKT/protein kinase B; Par6=partitioning-defective protein-6; aPKC=atypical protein kinase C.

The applied antibodies had been subjected to in-house validation by the manufacturer for IHC analysis on paraffin-embedded material. For the antibodies used in the study, see Table 1. For staining with fascin and NF-*κ*B p105, sections were deparaffinised with xylene and rehydrated with ethanol. Antigen retrieval was performed by placing the specimen in 0.01 mol l^−1^ citrate buffer at pH 6.0 and exposed to two repeated microwave heatings of 10 min at 450 W. The DAKO EnVision+System-HRP (DAB) kit was used for endogen peroxidase blocking. Primary antibodies for fascin and NF-*κ*B p105 were incubated for 30 min at room temperature. For staining with the remaining antibodies, the slides were transferred to the Ventana Benchmark, XT automated slide stainer (Ventana Medical System, Illkirch, France). The DAKO EnVision+ System-HRP (DAB) kit was used to visualise the antigens for all stains. This yielded a brown reaction product at the site of the target antigen. Tissue sections were incubated with primary antibodies recognising vimentin, E-cadherin, Par6, aPKC and TGF-*β*. Primary antibodies were incubated at 37°C (vimentin 24 min, E-cadherin 32 min, aPKC 28 min, Par6 52 min and TGF-*β* 28 min). As negative staining controls, the primary antibodies were replaced with the primary antibody diluent. Finally, all slides were counterstained with haematoxylin to visualise the nuclei. For each antibody, including negative controls, all TMA stainings were performed in a single experiment (Figure 1).

### Scoring of IHC

**Figure 2:**
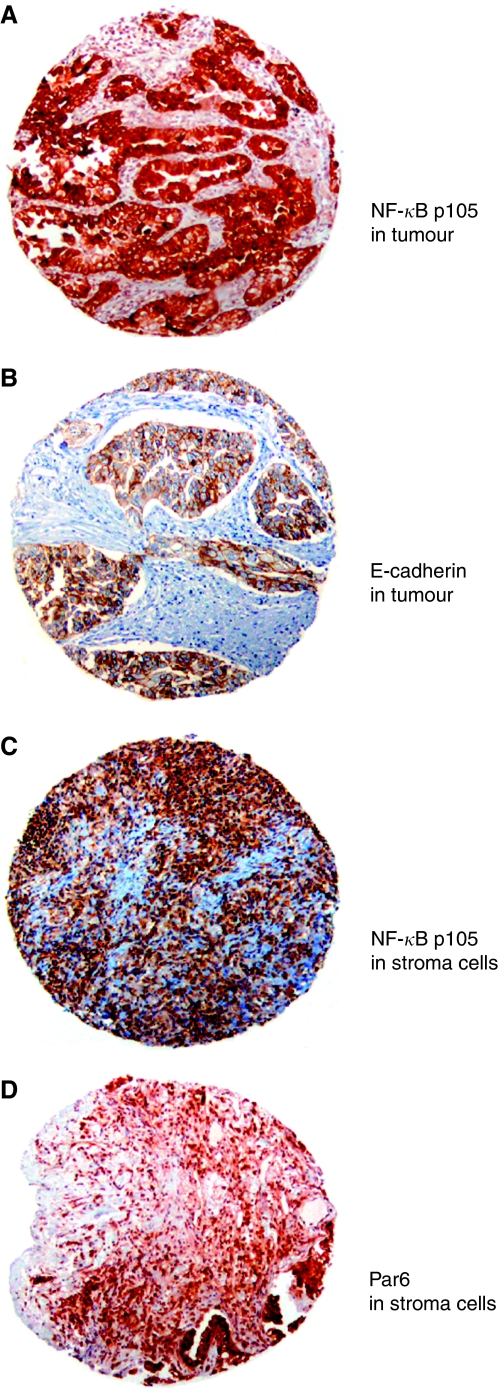
Immunohistochemical analysis of TMA of NSCLC representing different EMT markers including (**A**) NF-*κ*B p105 and (**B**) E-cadherin in tumour as well as (**C**) NF-*κ*B p105 and (**D**) Par6 in stromal cells. NF-*κ*B=nuclear factor-*κ*B; NSCLC=non-small-cell carcinoma; TMA=tissue microarray.

By light microscopy, representative viable tissue sections were scored semiquantitatively for cytoplasmic or membranous staining (Figure 2). The dominant staining intensity in both tumour epithelial cells and stromal cells was scored as 0=negative; 1=weak; 2=intermediate; and 3=strong. The cell density of the stroma was scored as 1=low density; 2=intermediate density; and 3=high density. All samples were anonymised and independently scored by two pathologists (SAS and KAS). In case of disagreement, the slides were re-examined and a consensus was reached by the observers. When assessing one variable for a given core, the observers were blinded to the scores of the other variables and to outcome. To evaluate the interobserver agreement with respect to IHC scoring, 100 consecutive tumour epithelial cell cores and tumour stroma cores, stained for two rabbit polyclonal markers (VEGF-C and VEGFR-3), were examined (Donnem *et al*, 2007). The mean correlation coefficient (*r*) was 0.95 (range 0.93–0.98) and was assessed for both antibodies in both tumour epithelial and stromal areas.

Mean score for duplicate cores from each individual was calculated separately in tumour epithelial cells and stroma, and a high expression in tumour epithelial cells was defined as score ⩾2. Stromal expression was calculated by summarising density score (1–3) and intensity score (0–3) before categorising into low and high expression. High expression in stroma was defined as score ⩾4.

### Statistical methods

All statistical analyses were performed using the statistical package SPSS (Chicago, IL, USA), version 14. The *χ*^2^ test and Fisher's Exact test were used to examine the association between molecular marker expression and various clinicopathological parameters. Univariate analysis was performed by using the Kaplan–Meier method, and statistical significance between survival curves was assessed by the log rank test. Disease-specific survival (DSS) was determined from the date of surgery to the time of lung cancer death. To assess the independent value of different pre-treatment variables on survival, in the presence of other variables, multivariate analysis was carried out using the Cox proportional hazards model. Only variables of significant value from the univariate analysis were entered into the Cox regression analysis. Probability for stepwise entry and removal was set at 0.05 and 0.1, respectively.

### Ethics clearance

The National Data Inspection Board and The Regional Committee for Research Ethics approved the study.

## Results

### Patient data

Demographical, clinical and histopathological variables are shown in Table 1. The median age was 67 years (range 28–85) and 75% of the patients were male individuals. The patient population of 335 cases represented the four major subtypes of NSCLC with 191 SCCs, 95 ACs, 31 LCCs and 18 BACs. Owing to nodal metastases or non-radical surgical margins, 18% (59 patients) received postoperative radiotherapy.

### Expression pattern and correlations with clinicopathological variables

Our IHC analyses included the phosphorylated NF-*κ*B p105, vimentin, E-cadherin, aPKC, Par6, fascin and TGF-*β*. All the investigated markers except E-cadherin were expressed in the cytoplasm of tumour epithelial cells. E-cadherin showed a membranous staining. On the basis of morphological criteria, pneumocytes in control cores from normal lung tissue, distant from the primary tumour, generally showed weak positive immunostaining.

In tumour stroma and in control cores, inflammatory cells (macrophages, lymphocytes, granulocytes and plasma cells) and endothelial cells frequently showed positive staining for NF-*κ*B p105, vimentin and Par6, whereas fibroblast-like cells only occasionally presented positive staining. No stromal cell staining was observed for E-cadherin, fascin, TGF-*β* and aPKC.

Expression of the investigated markers in tumour epithelial cells or stroma did not correlate with clinical performance status, vascular infiltration or histological subgroups. Expression of vimentin and Par6 in tumour epithelial cells correlated significantly (*r*=0.2, *P*<0.001), as did vimentin and tumour differentiation (*r*=−0.1, *P*=0.01). High expression of vimentin was seen in 61% of poorly differentiated tumours, 39% of moderately differentiated tumours and no expression was seen in well-differentiated tumours. Even among the patient group with poorly differentiated tumours, high tumour epithelial cell vimentin expression tended to correlate with poor survival (*P*=0.08). Further, a significant correlation was seen between tumour epithelial expression of aPKC and Par6 (*r*=0.2, *P*<0.001).

### Univariate analysis

**Figure 3:**
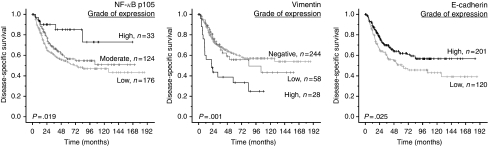
Disease-specific survival curves for tumour epithelial cell NF-*κ*B p105, vimentin and E-cadherin. NF-*κ*B=nuclear factor-*κ*B.

**Figure 4:**
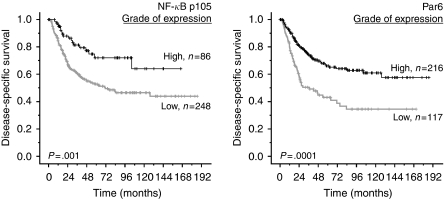
Disease-specific survival curves for stromal NF-*κ*B p105 and Par6. NF-*κ*B=nuclear factor-*κ*B; Par6=partitioning-defective protein-6.

In addition to the statistically significant clinical variables (Table 2), tumour epithelial cell expression of NF-*κ*B p105 (*P*=0.02), vimentin (*P*=0.001) and E-cadherin (*P*=0.03), and stromal cell expression of NF-*κ*B p105 (*P*=0.001) and Par6 (*P*=0.0001) were prognostic indicators for DSS in univariate analyses (Table 3; Figures 3 and 4). Stratifying the cases based on histology revealed that the significance of better survival for high tumour epithelial NF-*κ*B p105 and vimentin expression was limited to ACs (*P*=0.03 and *P*=0.0004, respectively) and stromal NF-*κ*B p105 and Par6 expression to SCCs (*P*=0.001 and *P*=0.0009, respectively). There was no significant association between DSS and tumour epithelial cell expression of aPKC (*P*=0.2), Par6 (*P*=0.7), fascin (*P*=0.4), TGF-*β* (*P*=0.1) or stromal cell expression of vimentin (*P*=0.3) (Table 3).

### Multivariate Cox proportional hazards analysis

All significant clinicopathological and molecular variables from the univariate analyses were entered into the multivariate analysis. Data are presented in Table 4. Tumour epithelial cell expression of NF-*κ*B p105 (*P*=0.001) and vimentin (*P*=0.005), stromal cell expression of NF-*κ*B p105 (*P*=0.007) and Par6 (*P*=0.0001), and the clinicopathological variables T stage (*P*=0.02), N stage (*P*=0.0001) and performance status (*P*=0.03) were all independent prognostic factors for survival. High tumour epithelial cell expression of E-cadherin (*P*=0.05), histological differentiation (*P*=0.05) and vascular infiltration (*P*=0.06) tended towards a statistical significance.

## Discussion

Using high-throughput TMA analyses, an established method for assessing protein expression across large sets of lung cancer tissues (Bremnes *et al*, 2002), we sought to determine the prognostic impact of a panel of seven molecular markers associated with tumorigenesis. High stromal expression of NF-*κ*B p105 and Par6 as well as high epithelial tumour cell expression of NF-*κ*B p105 showed an independent positive correlation with DSS. In contrast, vimentin expression in tumour epithelial cells was an independent negative prognostic indicator for DSS.

Immunohistochemistry as a method for detecting protein expression in paraffin-embedded tissue has been shown to be both highly sensitive and specific (Zafrani *et al*, 2000), yet the specificity of an immunohistochemical test would never exceed the specificity of the antibody provided by the manufacturer. Nevertheless, an additional possible source of error can still be the biological variation of protein expression in different areas of tumour tissue. Nonetheless, this source of bias can be reduced by increasing the number of examined tissue as in this study.

Stromal–epithelial interactions are considered critical for regulating tissue development and for the maintenance of tissue homoeostasis (Kass *et al*, 2007). Consequently, it seems essential also to study the tumour stroma and its different molecular markers to be able to understand the mechanism of tumour metastases. In this study, the term ‘stroma’ comprises all groups of non-epithelial cells and structures intervening between islands of tumour epithelial cells, that is mesenchymal cells (fibroblasts and fibroblast-like cells), leukocytes, macrophages, endothelial cells and extracellular matrix (ECM) including collagen. Investigating the stromal compartment can, however, be complicated as the stroma is not static. Cellular and ECM compositions evolve over time, adapting to changes related to the surrounding epithelial cells (Kass *et al*, 2007).

Stromal cells appear, in some instances, to support growth and motility of tumour cells (Costea *et al*, 2006), and in other instances to be part of the microenvironment in preventing tumour cell invasion, in concert with the assumed function of the immune system. Thus, stromal cells have rather complex and controversial roles during the ‘abnormal’ condition of tumorigenesis. Hence, it is of great interest that a high stromal expression of NF-*κ*B p105 and Par6 correlates favourably with patient survival.

Nuclear factor-*κ*B is a group of proteins that control inflammation, cell survival, transformation, proliferation, angiogenesis and apoptosis (Aggarwal, 2004). It is normally retained in the cytoplasm in an inactive state through interaction with inhibitor *κ*B (I*κ*B) (Karin *et al*, 2002). Degradation of the I*κ*B proteins results in the liberation of NF-*κ*B, allowing nuclear translocation and the activation of target genes, including Snail and Bcl-2 (Guarino, 2007). NF-*κ*B is activated in a range of human cancers and is assumed to promote tumorigenesis (Radisky and Bissell, 2007). Five mammalian NF-*κ*B proteins have been identified: p65 (RelA), NF-*κ*B1 (p50 and its precursor p105), NF-*κ*B2 (p52 and its precursor p100), c-rel and RelB (Ghosh *et al*, 1990). These bind to DNA as homo- or heterodimers.

Havard *et al* (2005) observed high levels of NF-*κ*B p105 precursor in cell lines derived from human cervical cancers associated with HPV16 infection and in keratinocytes transfected by oncogenes. In another study, Ishikawa *et al* (1998) observed that NF-*κ*B p105−/− mice showed abnormalities such as inflammation in lungs and liver, myeloid hyperplasia in bone marrow and splenomegaly (Weih *et al*, 1994). In a study that included 45 patients with NSCLC, Zhang *et al* (2006) found overexpression of NF-*κ*B p50 to indicate an unfavourable overall survival in NSCLC patients. To our knowledge, the prognostic significance of the precursor NF-*κ*B p105 in both tumour epithelial and stromal cells of NSCLC has hitherto not been reported. In our study, NF-*κ*B p105 expressions in both tumour epithelial and stromal cells were favourable independent prognostic indicators for survival.

Par6 and aPKC have emerged as central players in the regulation of cell polarity and the asymmetric division of cells (Etienne-Manneville and Hall, 2003). Par6 is an adapter protein that engages in many protein–protein interactions regulated to control cell polarity (Thiery and Huang, 2005). In previous cancer cell line studies, Regala *et al* (2005) categorised aPKC-iota as an oncogene in NSCLC. Through PubMed searches, we could not identify studies investigating the prognostic significance of Par6 and aPKC-iota expression in resected primary NSCLC tissues. In this study, we found high Par6 stromal cell expression to be associated with an improved prognosis, whereas tumour epithelial cell expression of Par6 and aPKC-iota did not show any prognostic relevance. These results indicate that an observed significance of protein expression in cell lines *in vitro* does not necessarily have the same impact *in vivo*. The interplay with the ECM may be essential in explaining the discrepancy of these results.

Cadherin-mediated cell–cell adhesion is considered a suppressor of cancer cell invasion *in vitro* (Behrens *et al*, 1993). Loss of E-cadherin has previously been attributed to an unfavourable prognostic significance in bladder cancer (Baumgart *et al*, 2007). Low E-cadherin expression in NSCLC tumours has been reported in several studies (Liu *et al*, 2001) to be associated with a more ‘aggressive’ behaviour of tumour epithelial cells and with a worse prognosis. Our results on E-cadherin expression in tumour epithelial cells are consistent with these findings.

We further examined the significance of fascin and vimentin expression in NSCLC. Fascin is an actin-bundling and crosslinking protein that binds to preformed filaments and regulates their organisation and stability. It is presumed to regulate cortical cell membrane protrusions (Kureishy *et al*, 2002). Its overexpression is proposed to increase the motility of epithelial cells (Adams, 2004). The available data on fascin expression and its significance in NSCLC are scarce. In a recent study by Pelosi *et al* (2003) in 220 stage I NSCLC patients, high tumour cell expression of fascin emerged as an independent predictor of lymph node metastases and poor survival. Our data did not, however, show any correlation between tumour expression of fascin and lymph node metastases or survival.

Vimentin is a structural protein from cells of mesenchymal origin (Leader *et al*, 1987). Its expression is higher in migratory epithelial cells and may contribute to the migratory and invasive phenotype of metastatic cells (Bindels *et al*, 2006). Significant correlations between high vimentin tumour cell expression and poor prognosis have previously been reported both in hepatocellular (Hu *et al*, 2004) and breast carcinoma (Dandachi *et al*, 2001). Consistent with the latter reports, we found high tumour epithelial cell vimentin expression to be an independent prognosticator for poor survival in resected NSCLC patients. In contrast, Pelletier *et al* (2001) in 113 resected NSCLC patients found no such correlation.

Transforming growth factor-*β* is a multifunctional cytokine that plays a central role in signalling networks regulating cell growth, differentiation, adhesion and apoptosis, and is one of the most potent and better-studied markers inducing mesenchymal phenotype in epithelial cells (Janda *et al*, 2002). There are indications that TGF-*β*-dependent loss of tight junctions is mediated through Par6 phosphorylation by the TGF-*β* receptor and is probably independent of Smad-mediated transcriptional responses (Ozdamar *et al*, 2005). In a recent study, von Rahden *et al* (2006) observed a correlation between high TGF-*β* tumour cell expression and a poor prognosis in oesophageal AC. In NSCLC tumours (Boldrini *et al*, 2000; Hasegawa *et al*, 2001; Saji *et al*, 2003), there are conflicting data regarding associations between TGF-*β* expression and survival. In this study, no significant association was seen between TGF-*β* tumour cell expression and overall survival.

In summary, we detected an unanticipated behaviour of tumour stromal cells and their impact on survival. Hence, the interaction between tumour stromal cells and tumour epithelial cells plays an important role in the process of cell invasion and metastases, yet many nuances of this interaction are still not clear. Our data give rise to additional investigation, which may provide the foundation for generating more effective therapeutic strategies not only in NSCLC but also in other malignancies.

## Figures and Tables

**Table 1:** Antibodies

**Antigen**	**Antibody**	**Clone**	**Catalogue no.**	**Source**	**Dilution**
NF-*κ*B p105	Rabbit monoclonal	178F3	4808S	Cell Signaling Technology (Danvers, MA, USA)	1 : 50
Vimentin	Mouse monoclonal	V9	790–2917	Ventana Medical Systems (Illkirch, France)	a
E-cadherin	Mouse monoclonal	ECH-6	760–2830	Cell Marque (Rocklin, CA, USA)	a
Par6	Rabbit polyclonal	H90	Sc-25525	Santa Cruz Biotechnology (Santa Cruz, CA, USA)	1 : 10
aPKC	Rabbit polyclonal	C20	Sc-216	Santa Cruz	1 : 100
Fascin	Mouse monoclonal	55K2	MAB3582	Chemicon International (Temecula, CA, USA)	1 : 25
TGF-*β*	Rabbit polyclonal	V	Sc-146	Santa Cruz	1 : 50

aPKC=atypical protein kinase C; NF-*κ*B=nuclear factor-*κ*B; Par6=partitioning-defective protein-6.

a Pre-diluted from the manufacturer.

**Table 2:** Prognostic clinicopathological variables as predictors for disease-specific survival in 335 NSCLC patients (univariate analysis; log-rank test)

**Characteristics**	**Patients (*n*)**	**Patients (%)**	**Median survival (months)**	**5-year survival (%)**	***P*-value**
*Age (years)*					
⩽65	156	47	104	57	0.62
>65	179	53	NR	58	
					
*Sex*					
Female	82	25	127	65	0.19
Male	253	75	84	55	
					
*Smoking*					
Never	15	5	19	43	0.13
Present	215	64	NR	60	
Previous	105	31	84	54	
					
*Performance status*					
Normal	197	59	NR	62	0.04
Slightly reduced	120	36	61	52	
In bed <50%	18	5	36	40	
					
*Weight loss*					
<10%	303	90	127	57	0.92
>10%	32	10	NR	57	
					
*Histology*					
SCC	191	57	NR	65	0.30
Adenocarcinoma	95	28	52	44	
BAC	18	5	NR	67	
LCC	31	9	84	54	
					
*Differentiation*					
Poor	138	41	48	48	0.001
Moderate	144	43	NR	64	
Well	53	16	NR	65	
					
*Surgical procedure*					
Lobectomy+wedge^a^	243	73	NR	61	0.0009
Pneumonectomy	92	27	35	46	
					
*Stage*					
I	212	63	NR	68	<0.0001
II	91	27	41	46	
IIIa	32	10	18	22	
					
*Tumour status*					
1	90	27	NR	75	0.002
2	218	65	84	52	
3	27	8	42	43	
					
*Nodal status*					
0	232	69	NR	66	<0.0001
1	76	23	37	43	
2	27	8	18	20	
					
*Surgical margins*					
Free	307	92	127	58	0.34
Not free	28	8	64	51	
					
*Vascular infiltration*					
No	284	85	NR	61	0.0005
Yes	51	15	25	35	
					
*Postoperative radiotherapy*					
No	276	82	NR	61	0.002
Yes	59	18	41	42	

BAC=bronchioalveolar carcinoma; LCC=large-cell carcinoma; NR=not reached; NSCLC=non-small-cell carcinoma; SCC=squamous cell carcinoma.

a Wedge, *n*=10.

**Table 3:** Tumour epithelial cell and stromal cell expression of markers associated with mesenchymal phenotype as predictors for disease-specific survival in 335 NSCLC patients (univariate analysis; log-rank test)

**Marker expression**	**Patients (*n*)**	**Patients (%)**	**Median survival (months)**	**5-year survival (%)**	***P*-value**
*NF-κB p105*					
Tumour					0.019
Low	176	52	NR	53	
Moderate	124	37	71	56	
High	33	10	NR	85	
Missing	2	1			
Stroma					0.001
Low	248	74	71	53	
High	86	25	NR	72	
Missing	1	1			
					
*Vimentin*					
Tumour					0.001
Negative	244	73	NR	62	
Low	58	17	84	66	
High	28	8	22	33	
Missing	5	2			
Stroma					0.297
Low	90	27	104	54	
High	242	72	NR	59	
Missing	3	1			
					
*E-cadherin*					
Tumour					0.025
Low	120	36	61	51	
High	201	60	NR	62	
Missing	14	4			
Stroma					
Negative for staining					
					
*Par6*					
Tumour					0.73
Low	169	51	NR	59	
High	162	48	84	57	
Missing	4	1			
Stroma					0.0001
Low	117	35	37	43	
High	216	64	NR	66	
					
*aPKC*	2	1			
Tumour					0.154
Low	54	16	NR	66	
High	278	83	84	56	
Missing	3	1			
Stroma					
Negative for staining					
					
*Fascin*					
Tumour					0.422
Low	96	28	29	53	
High	235	70	NR	60	
Missing	4	1			
Stroma					
Negative for staining					
					
*TGF-β*					
Tumour					0.128
Low	270	81	104	55	
High	61	18	NR	69	
Missing	4	1			
Stroma					
Negative for staining					

aPKC=atypical protein kinase C; NF-*κ*B=nuclear factor-*κ*B; NSCLC=non-small-cell carcinoma; Par6=partitioning-defective protein-6.

**Table 4:** Results of Cox regression analysis summarising significant independent prognostic factors

**Factors**	**Hazard ratio**	**95% CI**	***P*-value**
*Tumour status*			0.022^a^
1	1.000		
2	1.822	1.080–3.072	0.025
3	2.681	1.278–5.626	0.009
			
*Nodal status*			0.0001^a^
0	1.000		
1	1.942	1.237–3.048	0.004
2	2.874	1.566–5.276	0.001
			
*Performance status*			0.034^a^
Normal	1.000		
Slightly reduced	1.696	1.139–2.526	0.009
In bed <50%	1.298	0.503–3.350	0.590
			
*Differentiation*			0.053^a^
Poor	1.000		
Moderate	1.473	0.783–2.770	0.230
Well	0.875	0.459–1.669	0.685
			
*Vascular infiltration*			
No	1.000		
Yes	1.603	0.975–2.636	0.063
			
*NF-κB p105 tumour*			0.001^a^
High	1.000		
Moderate	8.986	2.821–28.629	0.0001
Low	7.117	2.182–23.214	0.001
			
*NF-κB p105 stroma*			
High	1.000		
Low	2.164	1.231–3.806	0.007
			
*Vimentin tumour*			0.005^a^
High	2.695	1.441–5.039	0.002
Moderate	0.892	0.529–1.504	0.667
Negative	1.000		
			
*E-cadherin tumour*			0.052^a^
High	1.000		
Low	1.467	0.997–2.158	
			
*Par6 stroma*			0.0001^a^
High	1.000		
Low	2.458	1.660–3.640	0.0001

CI=confidence interval.

a Overall significance as a prognostic factor.
